# Compound coastal marine–terrestrial heatwaves associated with humid-heat stress in Europe

**DOI:** 10.1038/s41598-025-32049-z

**Published:** 2025-12-12

**Authors:** Armineh Barkhordarian, Eline Brunet, Johanna Baehr

**Affiliations:** 1 Institute of Oceanography, Universität Hamburg, Hamburg, Germany; 2 Institut Universitaire Européen de la Mer (IUEM), Université de Bretagne Occidentale (UBO), Brest, France

**Keywords:** Climate sciences, Environmental sciences, Natural hazards, Ocean sciences

## Abstract

European coastal regions - home to dense populations, and climate-sensitive ecosystems - are vulnerable to the compounding effects of marine–terrestrial heatwaves, defined here as concurrent extreme heat events over land and adjacent ocean areas. Using satellite and station-based observations, we find a nonlinear and accelerating increase in exposure to these compound events across European coastlines over the past two decades, peaking at 78 days in the Mediterranean in 2022. Attribution analysis reveals that greenhouse gas (GHG) forcing accounts for 95% of the risk of the 2022 event, and the CESM1-LE simulations indicate that such an event would be virtually absent without GHG forcing, emphasizing the nonlinear escalation of risk as GHG emissions continue. We further demonstrate that simultaneous marine heatwaves in the adjacent ocean can amplify coastal terrestrial heatwave exposure by up to 3.5 times, transforming short-lived terrestrial heatwaves into prolonged episodes of extreme heat and humidity. During compound events, coastal regions exhibit a pronounced shift toward humid heatwave regimes, characterized by wet-bulb temperatures $> 25.5\, ^\circ$C and elevated specific humidity. Our finding reveals an amplifying effect of marine heatwaves on the persistence of coastal terrestrial heat extremes through enhanced moisture and heat coupling at the land–sea interface, underscoring the growing climate vulnerability of coastal populations.

## Introduction

Human influence, primarily through greenhouse gas emissions, has unequivocally warmed both the ocean and atmosphere on a global scale^1–4^. Consequently, marine heatwaves (MHWs) have increased in frequency, intensity, and duration across most ocean basins^5–8^, with profound socioeconomic consequences^9–12^. Similarly, hot extremes over land have risen even more rapidly than in the ocean, driving stronger, longer, and often record-breaking terrestrial heatwaves^3,13–17^. Statistical analyses have identified occurrences of marine heatwaves and terrestrial heatwaves simultaneously in some regions, including coastal Australia^18^, New Zealand^19,20^, Northwest Pacific^21^, and southeast China^22^. The study by Hu (2021)^23^ conducted a global analysis of 38 coastal cities and found that during marine heatwaves, nearby cities also experienced unusually elevated temperatures and humidity, particularly pronounced at higher latitudes. There is evidence that driving factors -such as atmospheric blocking - plays significant role in the concurrent occurrence of marine and terrestrial heatwaves^18,19,24^. In this study, we quantify compound coastal marine–terrestrial heatwaves in Europe, defined here as events in which a marine heatwave fully encompasses a terrestrial heatwave within paired coastal land and adjacent ocean grid cells. This paired-coast framework provides the first systematic assessment of compound heatwaves that directly links ocean and land extremes at the same coastline, thereby revealing their potential to intensify humid-heat stress in nearby European coastal communities.

Emerging evidence suggests that the ecological and economic consequences of compound marine–terrestrial heatwaves may surpass those associated with isolated heatwave events. For instance, in Western Australia a massive heatwave event affected during the austral summer of 2011 both terrestrial and marine ecosystems, leading to abrupt, synchronous, and multi-trophic ecological disturbances^25^. These disruptions included mortality, changes in demographics, and shifts in species distributions. Tree die-off and coral bleaching occurred concurrently in response to the heatwave, and were accompanied by terrestrial plant mortality, seagrass and kelp loss, and a population crash of an endangered terrestrial bird species, among other impacts^25^. Another high-impact compound event occurred during the summers of 2017/2018 in New Zealand^19^, where an unprecedented coupled ocean-atmosphere heatwave, covering an area of 4 million km^2^, persisted for the entire austral summer. This compound event led to a loss of 3.8 ± 0.6 km^3^ of glacier ice in the Southern Alps, marking the largest annual loss recorded since 1962, and caused substantial disturbances in marine ecosystems^19,20^. In semi-arid Mediterranean regions, dry compound extremes have been linked to enhanced wildfire activity through concurrent atmospheric heatwaves, drought, and marine heatwaves^26^.

European coastal regions are particularly vulnerable to compound marine–terrestrial heat extremes, as the additive - and potentially synergistic - impacts of these events on densely populated coastal areas could significantly amplify health risks. Over the eastern Mediterranean Sea, more than half of marine heatwaves co-occur with atmospheric heatwaves over the Sea^27^, and such concurrence has been shown to intensify marine heatwaves^28^ through air–sea heat flux changes^29^. The remarkable atmospheric heatwave and marine heatwave events in the Mediterranean in 2003 have been the focus of several studies, which noted that elevated SST in the Mediterranean strengthened the European terrestrial heatwave after it began, although they did not contribute to its initiation^30,31^.

Previous studies have shown that atmospheric heat extremes can reinforce marine heat anomalies, particularly in the Mediterranean, but these assessments have largely been conducted at broad regional scales^27–29^ or by comparing land and ocean events independently^30,31^. As a result, they do not resolve how heatwave interactions unfold at specific coastlines. Here, we identify and quantify compound coastal marine–terrestrial heatwaves, defined as terrestrial heatwaves that are fully encompassed by concurrent marine heatwaves at paired coastal land and adjacent ocean grid cells. By using paired coastal points, we quantify not only the co-occurrence probability but also the local impacts on inland humid heat stress – an aspect that has not been addressed in previous studies.

This study addresses three primary objectives. First, we use satellite-derived daily sea surface temperature (SST; OISST^32^) and station-based daily 2m temperature ($T_{\text {2m}}$, E-OBS^33^) to examine the co-occurrence rates of marine and terrestrial heatwaves along European coastlines. We further quantify the role of coastal marine heatwaves in modulating terrestrial heatwave exposure along the European coastlines. Second, we assess the contribution of greenhouse gas (GHG) forcing to the observed increase in exposure to compound marine–terrestrial heat extremes. To this end, we apply extreme event attribution techniques^15,34^ using single-forcing experiments from the Community Earth System Model Large Ensemble (CESM1-LE^35^). Quantifying the extent to which GHG forcing contributes to the exposure time of extreme events is a key challenge spanning climate science, ecology, economics, and public health.

Finally, we investigate the intensity of coastal humid-heat stress during compound marine–terrestrial events. Coastal heatwaves, characterized by elevated humidity, can significantly exacerbate the effects of extreme temperature on human health. Humid-heat stress is evaluated using two indicators: wet-bulb temperature, and near-surface specific humidity. A transition toward humid heat extremes is evident during compound events, where rising wet-bulb temperatures and specific humidity jointly signal a new class of coastal heatwave conditions. Our findings underscore the urgent need for integrated risk assessments that account for the compound nature of heat stress and its anthropogenic drivers, and for the development of targeted, region-specific adaptation strategies across Europe’s most vulnerable coastal zones.

## Results

### Detection of compound coastal marine–terrestrial heatwaves over Europe

**Fig. 1:**
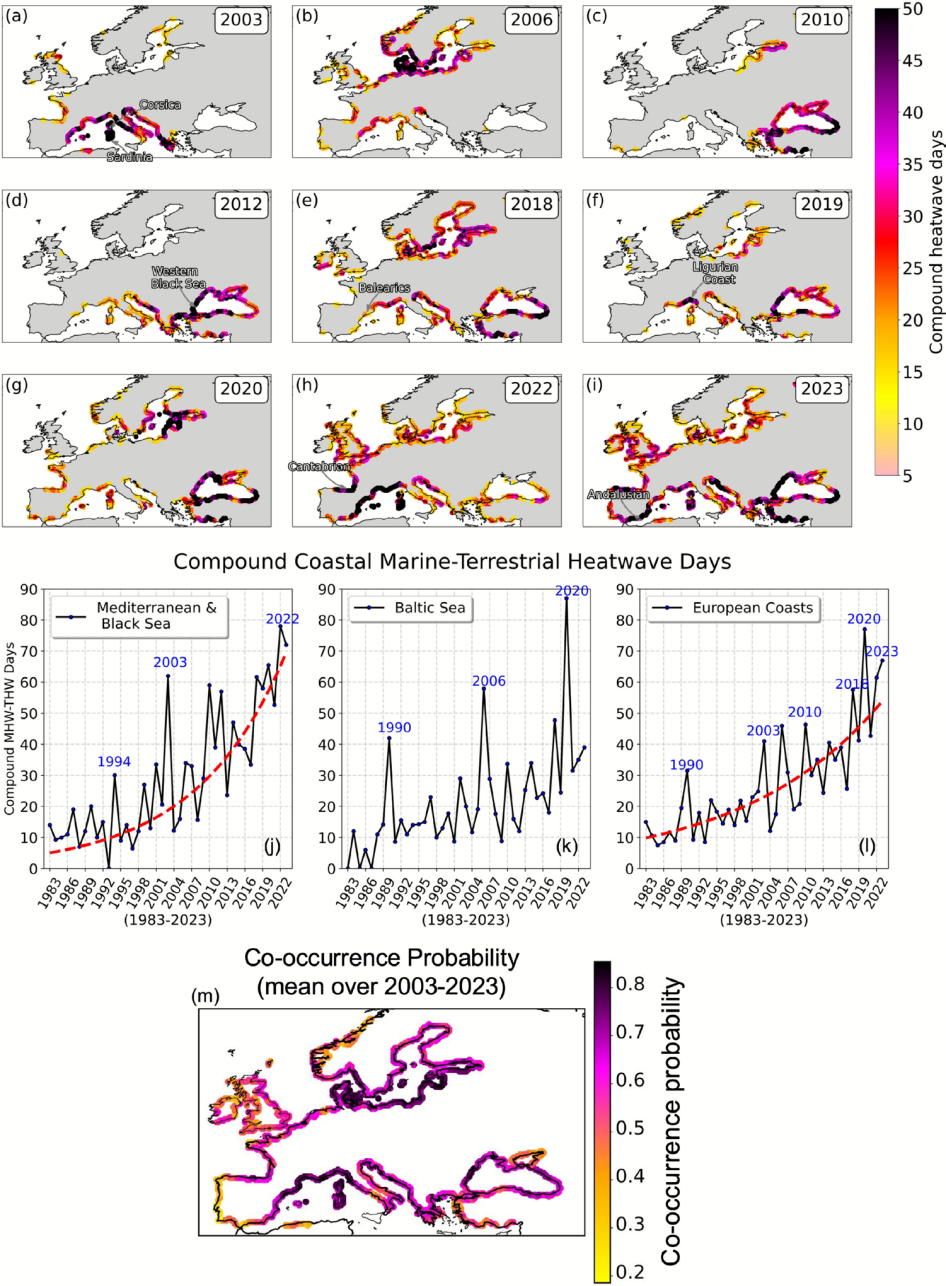
**a–i** Observed annual spatial distribution of compound marine–terrestrial heatwave days, based on OISST and E-OBS observational records. These results are limited to areas where E-OBS data coverage is available for terrestrial temperatures. **j–l** Temporal evolution of compound marine–terrestrial heatwave days from 1983 to 2023 along the Mediterranean region (including the Black Sea), Baltic Sea coasts, and all European coastlines. **m** Spatial distribution of co-occurrence probability, defined as the fraction of compound coastal marine–terrestrial heatwave days relative to all terrestrial heatwave days. Values range from 0 (no terrestrial heatwave days were compound) to 1 (all terrestrial heatwave days were compound) over the period 2003–2023.

The observed record, based on OISST^32^ satellite data and E-OBS^33^ station-based data, reveals a clear and increasingly rapid rise in both the exposure time and spatial extent of compound marine–terrestrial heatwave events over the past four decades along European coastlines, restricted to regions covered by E-OBS observational data. Co-occurring heatwave days are defined as instances when a marine heatwave fully encompasses a terrestrial heatwave within paired coastal land and adjacent ocean grid cells. These years – 2003, 2006, 2010, 2012, 2018, 2019, 2020, 2022, and 2023 – are highlighted in Fig. 1a–i. The Mediterranean arc, spanning the Catalan coast to the Gulf of Lion-including the Balearic Islands, Sardinia, Corsica, and the coastlines of eastern Spain, southern France, and western Italy - emerges as a hotspot for compound coastal heatwaves.

In line with observed records from mooring measurements^36–38^, coastal marine heatwaves were observed over the Mediterranean & Black Sea almost every year over the last decade. All years from 2010–2023 experienced compound heatwaves in several locations of the coastal Mediterranean & Black Sea (see the full time series from 1984 to 2023 in Supplementary Fig. S1). This suggests that not only are individual marine and land heatwaves becoming common^39^, but simultaneous coastal oceanic and terrestrial heatwaves are occurring more frequently. Notably, recent years such as 2022 (Fig. 1h) and 2023 (Fig. 1i) exhibit a substantial rise in exposure, measured as the total number of compound heatwave days per year. In 2023, several regions-including the Andalusian coast (Spain), the Cantabrian Coast, the western Black Sea coast, western Greece, and the Turkish Mediterranean coastline - experienced more than 50 days of compound coastal heatwave conditions (Fig. 1i).

The time series over 1983 to 2023 exhibit a robust upward trend in compound days over the four-decade period, with inter-annual variability influenced by extreme years such as 2003, 2006, 2018, 2020, and 2022 (Fig. 1j–l). The red trend lines indicate a steepening trend, especially in the last two decades, emphasizing the accelerating impact of climate change on European coastal heat extremes. In particular, the Mediterranean & Black Sea shows a strongly nonlinear increase, especially after the early 2000s. While the region experienced a moderate number of compound days in the 1980s and 1990s (typically fewer than 20 days), this number increased sharply after 2003, exceeding 62 days in recent peak years and reaching nearly 78 days in 2022 and 72 days in 2023 (Fig. 1j).

The time series for the broader European coastline similarly reveals a sharp increase in compound heatwave days, as highlighted by a nonlinear trend line (Fig. 1l). By 2022, the number of compound days reached nearly 77 – more than three times the early 21st-century baseline of fewer than 20 days. The curvature of these trends, illustrated by the red dashed fit lines in Fig. 1j and l, suggest an non-linear accelerating rate of compound coastal marine–terrestrial heatwave occurrences.

To understand where compound marine–terrestrial heatwaves are most prevalent, we analyze the spatial distribution of co-occurrence probability - defined as the ratio of compound days to terrestrial heatwave days - averaged over 2003–2023 along European coastlines covered by E-OBS observational data (Fig. 1m). The resulting patterns reveal regional hot-spots and highlight the varying strength of ocean–land heatwave coupling across different basins. The highest co-occurrence probabilities (>  0.8), indicating regions where more than 80% of terrestrial heatwave days coincide with marine heatwaves, are observed along the western Mediterranean coasts and around Sardinia and Corsica. These areas are characterized by shallow waters and frequent atmospheric stagnation, which promote the buildup of heat and humidity^40–42^. Similarly high co-occurrence values also appear along parts of the southern Baltic Sea coastline, including northern Poland, western Lithuania, and Latvia. Moderate concurrence probability of  0.6–0.8, implying that 60–80% of terrestrial heatwaves co-occur with marine heatwaves, are widespread across much of the Mediterranean Basin, including the Black Sea coasts (Fig. 1m). In contrast, Atlantic coastlines exhibit lower co-occurrence fractions (0.2–0.4), indicating a weakern – though still notable – coupling between marine and terrestrial heat extremes (Fig. 1m). These patterns reinforce the notion that marine heatwaves are playing a growing role in modulating terrestrial extreme heat events, particularly in enclosed and semi-enclosed basins like the Mediterranean and Baltic Seas.

### Quantifying the role of marine heatwaves in modulating terrestrial heatwave exposure

**Fig. 2:**
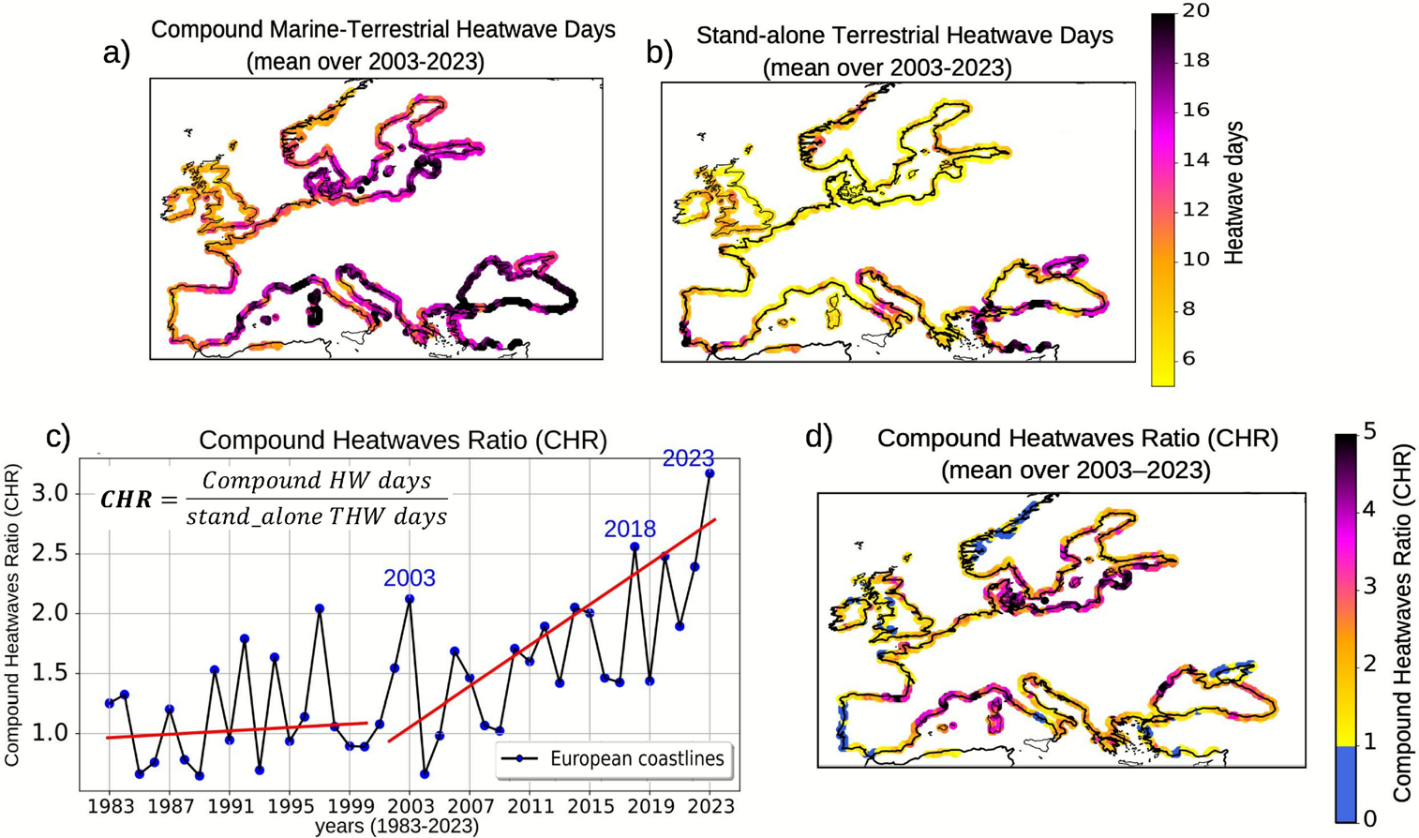
**a** Observed annual number of compound marine–terrestrial heatwave days along coastal grid cells, averaged over 2003–2023, based on OISST and E-OBS observational records, restricted to areas where E-OBS data coverage is available for terrestrial temperatures. **b** Number of stand-alone coastal terrestrial heatwave days, defined as terrestrial events occurring in the absence of simultaneous marine heatwave in adjacent ocean grid cells, averaged over 2003–2023. **c** Temporal evolution of the Compound Heatwave Ratio (CHR), defined as the ratio of compound marine–terrestrial heatwaves to stand-alone terrestrial heatwaves during 1983–2023. This metric quantifies how frequently compound events occur relative to stand-alone terrestrial events. **d** Spatial distribution of CHR along the coasts, averaged over 2003–2023.

A spatial comparison between stand-alone terrestrial heatwave days – defined as events occurring without a simultaneous marine heatwave in the adjacent ocean cell – and compound heatwave days reveals a growing predominance of compound events in shaping extreme heat exposure along coastal regions (Fig. 2a, b). The mean number of stand-alone terrestrial heatwave days during 2003–2023 remains below 10 in most coastal areas (Fig. 2b). In contrast, the frequency of compound marine–terrestrial days exceeds 20 days in several southern and eastern European coastal regions, indicating a pronounced dominance of compound heatwave occurrences –particularly along the western Mediterranean and the eastern Baltic coasts (Fig. 2a). To assess the role of marine heatwaves in modulating terrestrial heatwave exposure, we analyze the ratio of co-occurring heatwave days to stand-alone terrestrial heatwave days for each grid cell along the European coastline. We refer to this metric as the Compound Heatwaves Ratio (CHR; Eq. 1 in Methods; Fig. 2c, d).

In the early record (1980s–1990s), CHR fluctuates around 1, meaning terrestrial heatwaves were mostly independent, and occur in isolation and in conjunction with marine heatwaves at the same frequency (Fig. 2c). Since 2003, however, the ratio has consistently exceeded one, suggesting that terrestrial heatwave exposure time tends to increase in the presence of concurrent marine heatwaves. For 2023, the CHR peakes at 3.5, indicating that terrestrial heatwave exposure was 3.5 times greater when a marine heatwave occurred simultaneously. The post-2003 acceleration in CHR aligns with intensification of marine heatwaves, pointing to an increasing influence of land–sea interactions in shaping extreme heat conditions along European coastlines.

The spatial distribution of the compound heatwaves ratio, averaged over 2003–2023, reveals a pronounced dominance of compound heatwave occurrences across several European coastal regions (Fig. 2d). CHR values exceeding 3 are evident along the western Mediterranean coastline-including southern Spain, southern France, and western Italy-indicating that terrestrial heatwave exposure in these regions is more than three times higher when co-occurring with marine heatwaves than when occurring independently. Elevated CHR values (2–3) are also observed along parts of the Adriatic Sea, Aegean Sea, and the eastern Baltic coast. These results imply that terrestrial heatwaves are becoming more prolonged when co-occurring with nearby marine heatwaves. In contrast, much of the Atlantic coastline and northern European coasts exhibit lower CHR values $(<1.5)$, suggesting a weaker coupling between marine and terrestrial heatwave events in these areas.

In the following analysis, we utilize an event-attribution technique to assess the extent to which greenhouse gas forcing contributes to the exposure time of compound marine–terrestrial heatwaves along the European coastline.

### Attribution of compound heatwave exposure to greenhouse gas forcing

We use an extreme event attribution technique^43^ to identify the fraction of the likelihood of compound marine–terrestrial heatwave exposure that is attributable to GHG forcing^7,8^. We quantify the co-occurrence rates of terrestrial and marine heatwaves along the European coastline as simulated in CESM1-LE by analyzing paired coastal land $T_{\text {2m}}$ and adjacent ocean SST data. We estimate the probabilities of compound heatwaves exposure occurring in the presence and absence of GHG forcing. These probabilities are calculated for both actual (ALL-forcing) and counterfactual (fixed GHG forcing) scenarios. The estimated probabilities are used to calculate two event-attribution metrics, namely the fraction of attributable risk (FAR, Eq. 2 in Methods) and Probability Ratio (PR, Eq. 3 in Methods). A summary of the FAR and PR values corresponding to the annual number of compound heatwave days for three selected observed events over the Mediterranean – 2003 (62 days), 2022 (78 days), and 2023 (72 days) – is presented in Table 1.

**Fig. 3:**
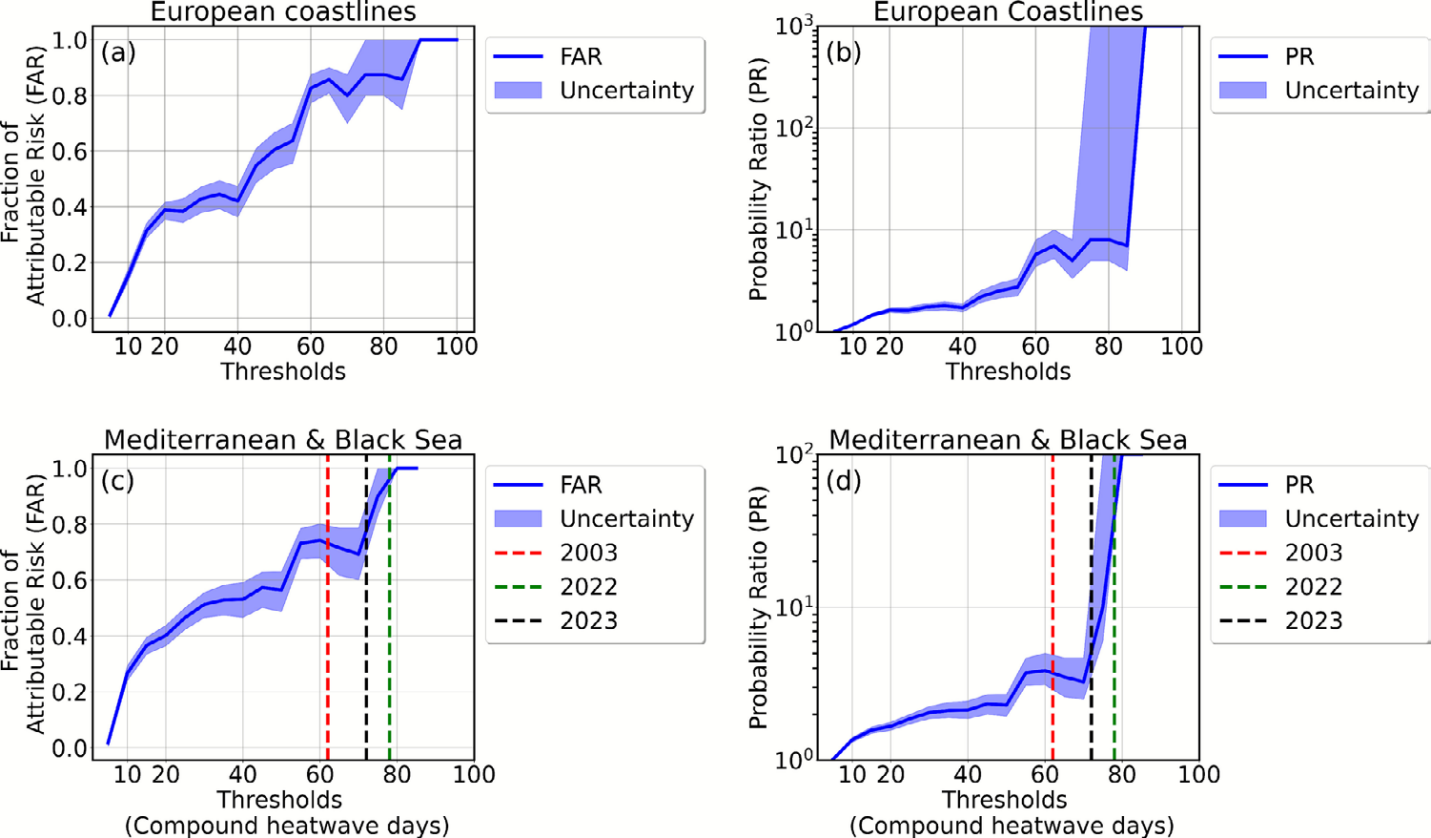
Attribution metrics: Fraction of Attributable Risk (FAR; blue curves) and Probability Ratio (PR; blue curves) for compound coastal marine–terrestrial heatwave days: **a, b** show results for the European coastlines, and **c, d** for the Mediterranean & Black Sea. The thresholds shown on the x-axis represent values of compound heatwave days. These threshold values are used in the calculation of the corresponding probabilities displayed on the y-axis. Dashed vertical lines in panels **c** and **d** indicate observed exposure durations for compound events over the Mediterranean & Black Sea in 2003 (62 days, red), 2022 (78 days, green), and 2023 (72 days, black). Shaded areas denote uncertainty ranges, estimated using a non-parametric bootstrap resampling approach.

The Fraction of Attributable Risk (FAR) curve, which quantifies the probability that GHG forcing is a necessary cause of an event, saturates at 1.0 when exposure to compound heatwaves exceeds 90 days along the European coastlines. This indicates that any compound event with exposure beyond this threshold would not occur without GHG forcing, with 99% probability (Fig. 3a). The corresponding Probability Ratio (PR) diverges to infinity, further supporting that such extreme compound events would be virtually absent in a climate without GHG forcing (Fig. 3b). At lower thresholds, the uncertainty range (blue uncertainty band) is narrow, meaning the estimates are more robust. At higher thresholds, the uncertainty widens, likely due to: Fewer extreme events, leading to larger sampling variability.

Over the Mediterranean & Black Sea, GHG forcing has increased the probability of an event with 62 days of exposure – such as the 2003 eventby a factor of 4 (PR = 4; 5–95% CI: 2.8–7.2). The corresponding fraction of attributable risk (FAR) is 0.72 (5–95% CI: 0.64–0.80), indicating that approximately 72% of the risk of occurrence can be attributed to GHG forcing (Fig. 3c, d, Table. 1 5). In other words approximately seven out of ten occurrences of extremes would not have happened without GHG forcing. The FAR value for the 2023 event, with 72 days of exposure, is 0.78 (5–95% CI: 0.7–0.88), indicating that such an event cannot be solely attributed to natural variability. GHG forcing accounts for approximately 78% of the risk of occurrence, serving as a necessary cause of the observed event. FAR values approaching 1 (100%) imply that GHG forcing is a necessary cause of the event.

The contribution of GHG forcing becomes even more pronounced in the case of the 2022 event. The FAR value for a compound event with 78 days of exposure - such as the 2022 event- is 0.95 (5–95% CI: 0.93–1.0) (Fig. 3c). This indicates that, on average, 95% of the risk of such an event can be attributed to GHG forcing. In other words, the CESM1-LE model suggests that the probability of the 2022 event occurring in a world without GHG forcing is less than 5% (5–95% CI: 7–0%). The corresponding probability ratio for the 2022 event ranges from $\sim 6$ to infinity (Fig. 3d; Table. 1). Here, PR = [6 – $\infty$) indicates that the minimum risk is about six-fold higher in the factual climate, while the upper bound diverges towards infinity because such an extreme compound events would have been virtually absent in a climate without GHG forcing. This further underscores the potential to mitigate such extremes and their associated impacts through reductions in GHG emissions.

### Return period shifts of coastal marine, terrestrial, and compound heatwaves due to GHG forcing

To understand the role of greenhouse gas (GHG) forcing in driving the observed nonlinear acceleration of compound coastal heat extremes, we assess how GHG emissions reshape the return periods of marine, terrestrial, and compound marine–terrestrial heatwaves. Specifically, we compare return periods under historical (ALL forcing) and fixed-GHG (FixGHG) scenarios in the CESM1-LE model, focusing on 5-, 10-, 20-, 50-, and 100-year events. An event with an X-year return period (a so-called X-year event) is expected to occur once every X years on average.

**Fig. 4:**
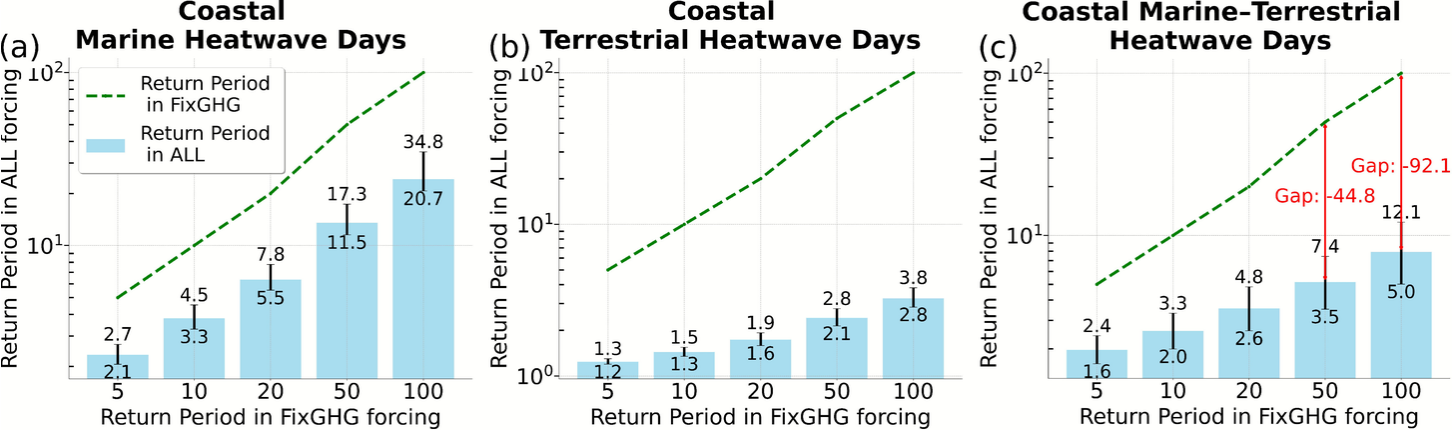
Return periods of heatwave days over the Mediterranean & Black Sea for **a** coastal marine, **b** coastal terrestrial, and **c** compound marine–terrestrial events. Light blue bars represent the return periods under the ALL forcing scenario, which includes all anthropogenic and natural forcings, while the green dashed line represents return periods under the FixGHG scenario, where greenhouse gas concentrations are held constant to isolate their effect. Annotations labeled “Gap” quantify the differences in return periods between the two scenarios. Error bars indicate the 2.5–97.5% confidence intervals estimated via a non-parametric bootstrap resampling approach.

Return periods of coastal marine heatwaves are consistently shorter (high in probability) under ALL forcing than under FixGHG, especially for high-threshold events (Fig. 4a). This indicates a clear influence of anthropogenic GHG emissions. For instance, a coastal marine heatwave with a 50- and 100-year return periods under FixGHG occurs approximately every 11.5 and 20.7 years, respectively, under ALL forcing – highlighting a marked increase in their frequency due to GHG forcing.

An even more dramatic response is seen for coastal terrestrial heatwaves (Fig. 4b). A 100-year event under FixGHG occurs every 3.2 years [2.8–3.8] under ALL forcing, corresponding to a  31-fold [26–36] increase in likelihood. Notably, return periods under ALL forcing remain nearly flat across the 5–100-year range: events expected once every 5, 10, 20, 50, or 100 years in a world without GHG forcing now occur approximately every 1.2, 1.4, 1.7, 2.4, and 3.2 years, respectively, under GHG forcing (Fig. 4b). This compression of recurrence intervals suggests a near-permanent shift toward persistent extreme heat over land. Given the humid nature of coastal regions, such intensified events can significantly exacerbate the effects of extreme temperature on human health.

The response is also strong for compound marine–terrestrial heatwave days (Fig. 4c), where GHG forcing leads to return period reductions-equivalent to increases in occurrence probability-of up to 92 years. At the 100-year FixGHG threshold, the same compound event recurs every 8 [5–12] years under ALL forcing. This amplification is visualized by the widening gap between the two return period curves and quantified by the red vertical lines labeled “Gap” in Fig. 4c. Notably, the increase in exposure time is nonlinear: the rarer the event under a FixGHG scenario, the greater its frequency boost under GHG forcing. This reflects a disproportionate rise in the occurrence of the most extreme compound events.

In summary, these findings demonstrate that GHG-driven climate change not only shifts the distribution of heat extremes but also steepens its upper tail – particularly for compound extremes - leading to a nonlinear acceleration in the occurrence of rare, high-impact events. The compound nature of these events – simultaneous extremes in both oceanic and terrestrial systems – underlines the elevated risk posed by anthropogenic climate change, with severe implications for coastal ecosystems, infrastructure, and human health.

**Table 1:** Attribution of compound coastal marine–terrestrial heatwave exposure time to GHG forcing. Shown are the fraction of attributable risk (FAR) and the probability ratio (PR) for three extreme compound events (2003, 2022, and 2023) over the Mediterranean & Black Sea. Brackets indicate 5–95% confidence intervals. The upper bound of the PR for 2022 event is infinite, reflecting a probability of zero in the counterfactual climate without GHG forcing.

Date of event	Threshold	FAR	PR
2003	62 days	0.72 [0.64 – 0.8]	4.1 [2.8 – 7.2]
2022	78 days	0.95 [0.93 – 1.0]	[6 – $\infty$)
2023	72 days	0.78 [0.7 – 0.88]	8 [4 – 10]

### Intensification of thermal-humidity extremes during compound events

The Mediterranean Sea is a key source of atmospheric moisture in the region, influencing both offshore and onshore humidity through synoptic-scale circulation and sea breeze dynamics^44–47^. As the basin warms, this moisture contribution is intensifying. Furthermore, enhanced land–sea thermal contrasts under greenhouse warming are expected to amplify these dynamics^48^, supporting the inland propagation of moisture from the warming sea.

**Fig. 5:**
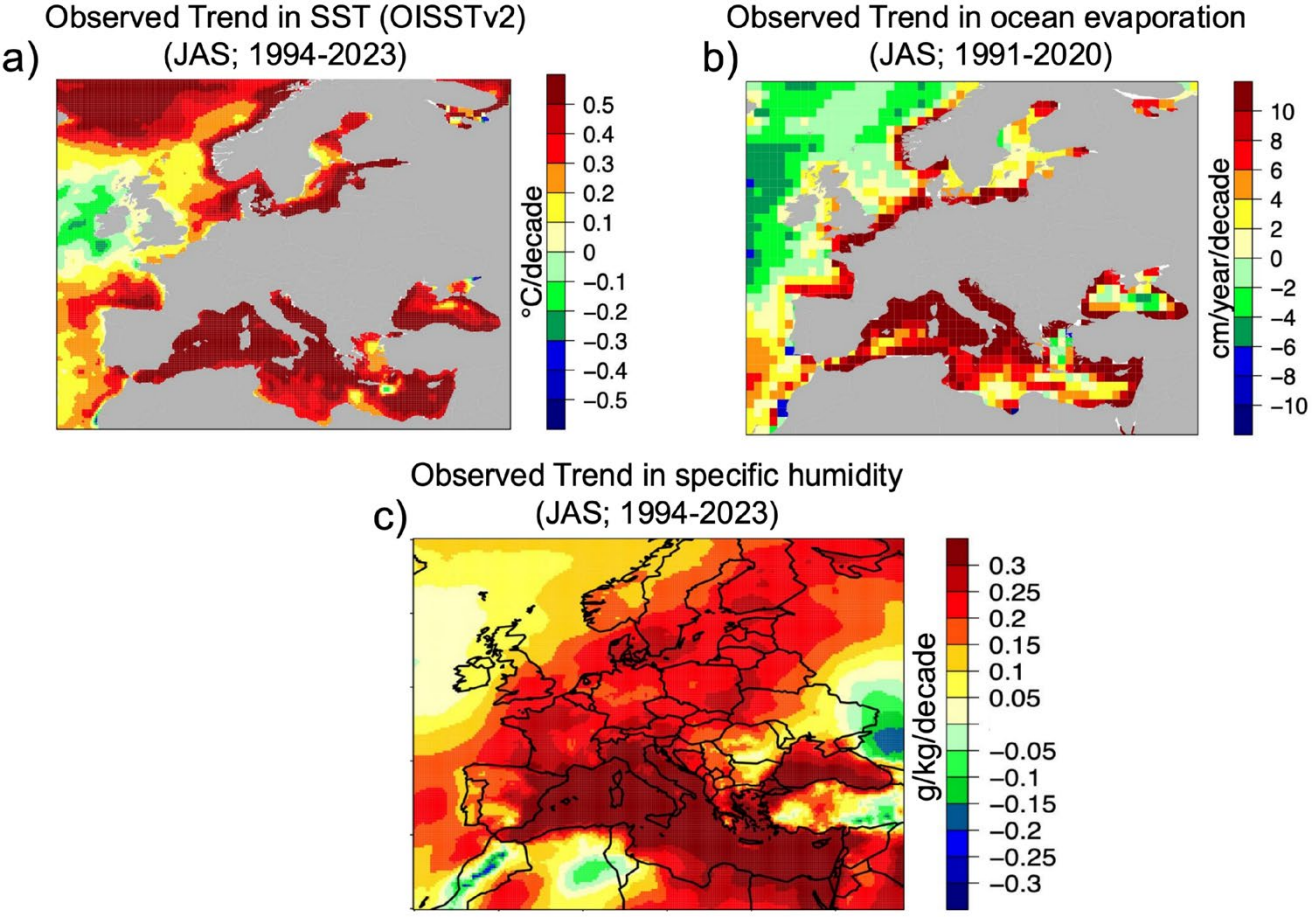
**a** Linear trend in satellite-based sea surface temperature (OISSTv2) during July–September (JAS) over the period 1994–2023, expressed in $^\circ$C per decade. **b** Linear trend in ocean evaporation from OAFlux data set during JAS for the period 1991–2020, expressed in cm/year per decade. **c** Linear trend in surface specific humidity from ERA5 during JAS over 1994–2023, expressed in g/kg per decade. All trends are computed using ordinary least squares regression.

Over the period 1994–2023, Mediterranean Sea surface temperatures – derived from the NOAA OISSTv2^32^ dataset –have increased by up to $0.5 ^\circ$C decade^-1^ (Fig. 5a). On 13 August 2022, the Balearic Islands region recorded the highest spatially averaged satellite SST since 1982, reaching $29.2 ^\circ$C – an anomaly of $3.3 ^\circ$C relative to the 1982–2015 climatology^36^. This exceptional marine warming is reshaping land–sea temperature gradients and strengthening coastal processes such as sea breezes, which can extend over a horizontal range of 100–150 km inland^49^ and play an important role in inland transport of moisture^50^. Concurrently, ocean evaporation has intensified, with localized increases exceeding 10 cm year^-1^ decade^-1^ during summer (JAS), based on OAFlux^51^ data (Fig. 5b). This enhanced surface moisture flux has contributed to a substantial rise in specific humidity over southern Europe and adjacent coastal zones, with trends reaching 0.3 g kg^-1^ decade^-1^ in summer, as calculated from ERA5^52^ reanalysis (Fig. 5c). Together, these trends provide strong observational evidence that warming of the Mediterranean Sea is amplifying atmospheric moisture availability, creating favorable conditions for elevated wet-bulb temperatures and the occurrence of humid heatwaves inland.

**Fig. 6:**
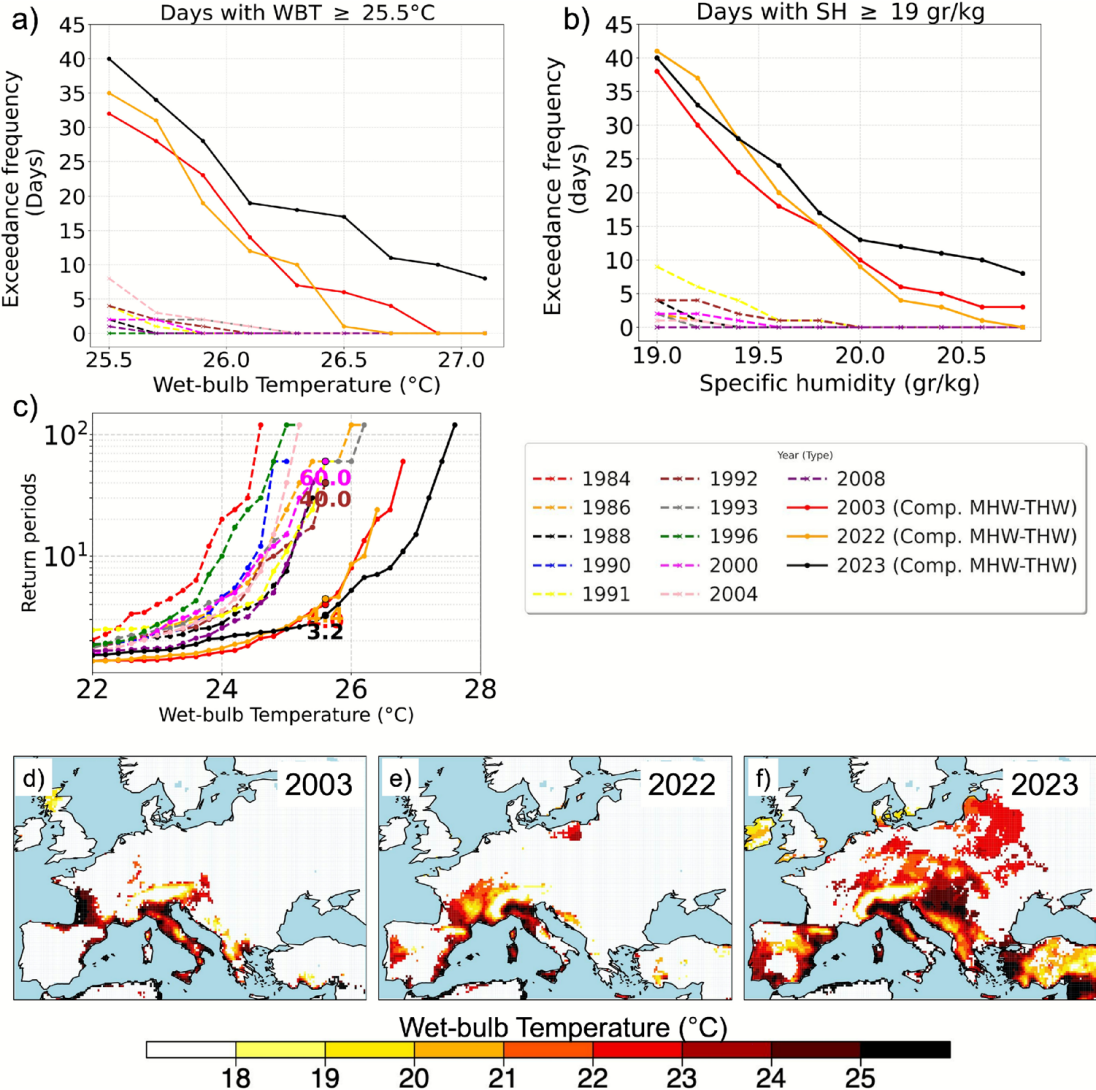
Annual number of days from ERA5 reanalysis during which: **a** Wet-bulb temperature exceeded $25.5 ^\circ$C (WBT $\ge$ $25.5 ^\circ$C) and **b** Specific humidity exceeded 19 g/kg (SH $\ge$ 19 gr/kg). Values represent spatial averages over land grid cells located up to 100 km inland from the Mediterranean coast, for years with compound coastal marine–terrestrial heatwaves (2003, 2022, 2023) compared to 11 non-compound years. The selection of non-compound years are based on the detection results shown in Supplementary Figure S1. WBT $\ge$ $25.5^\circ$C indicates unsafe heat stress per ISO 7243, and SH $\ge$ 19 g/kg marks highly humid conditions that exacerbate thermal discomfort and wet-bulb stress. **c** Return periods (in years) of extreme wet-bulb temperatures in the range of $22-28{^{\circ }}$C. **d–f** Spatial maps of maximum wet-bulb temperature during compound coastal marine–terrestrial heatwaves in 2003, 2022, and 2023.

To assess the frequency and severity of humid-heat stress along the coastline we use wet-bulb temperature (WBT), which reflects the combined effects of heat and humidity, and is an effective metric for characterizing extreme humid heat stress^53–55^. We analyzed the occurrence of wet-bulb temperature (WBT) exceedances above $25.5\, ^\circ$C average over land grid cells located up to 100 km inland from the Mediterranean coast. We utilize two complementary approaches: (1) exceedance frequency (WBT $\ge$ $25.5\, ^\circ$C) measured in days per year (Fig.6a), and (2) return periods, measured in years, over the 90-day summer season (June–August, JJA). Daily maximum WBT is estimated from ERA5 reanalysis using maximum temperature , dew point temperature, and surface pressure. The chosen WBT threshold is based on the ISO 7243 standard, which identifies WBT values above $25 ^\circ$C as unsafe for heavy outdoor labor without heat mitigation measures^56,57^. In addition, we analyzed the exceedance frequency of surface specific humidity (SH) above 19 g/kg (SH $\ge$ 19 g/kg). This threshold represents very high moisture content in the lower atmosphere and corresponds to conditions where elevated humidity exacerbates heat stress, reduces evaporative cooling efficiency, and drives wet-bulb temperatures toward hazardous levels. The selection of both compound and non-compound years is based on our detection results for 1984–2023 shown in Supplementary Figure S1.

The results show that compound years - 2003, 2022, and 2023 - exhibit a substantially higher frequency of days with wet-bulb temperature (WBT) $\ge$ $25.5\, ^\circ$C and specific humidity (SH)$\ge$ 19 g/kg, averaged over land grid cells located up to 100 km inland from the Mediterranean coast, compared to non-compound years (Fig. 6a, b). Notably, 2023 consistently records the highest number of extreme WBT days. The number of days exceeding the WBT $\ge$ $25.5\,^\circ$C threshold reaches  40 days in 2023, compared to fewer than 5 days in the selected 10 non-compound years. A similar pattern emerges for specific humidity, revealing a pronounced intensification of joint heat–humidity stress. In particular, 2022 and 2023 show frequent exceedance of SH $\ge$ 19 g/kg, highlighting exceptionally moist conditions that are largely absent during non-compound years (Fig. 6b).

The return period analysis highlights clear differences in the frequency of extreme humid-heat conditions between years with and without compound marine–terrestrial heatwave events (Fig. 6c). In compound years (e.g., 2003, 2022, 2023), return periods for high WBT thresholds (e.g., $> 26\, ^\circ$C) are substantially shorter, often falling below 5 years, indicating that such extreme humid-heat events have become more frequent. Particularly in 2023, the return period for WBT values approaching $26\, ^\circ$C drops to nearly 3–4 years, suggesting an exceptionally elevated frequency of hazardous humid-heat conditions. In contrast, the non-compound year 2000, for instance, shows return periods of about 60 years for the same thresholds, emphasizing the role of compound MHW-THW events in amplifying thermal stress. These results underscore that compound events do not merely raise mean conditions, but fundamentally alter the frequency distribution of extremes, shifting high-stress WBT levels from rare (decadal) to near-annual occurrences. This has direct implications for human health, infrastructure stress, and coastal climate adaptation strategies.

The spatial patterns confirm widespread intensification of wet-bulb extremes and highlights the spatial distribution of average WBT during compound MHW-THW days in 2003, 2022, and 2023 (Fig. 6d–f). The geographic extent and magnitude of WBT anomalies intensify markedly in recent years. In 2023, large swaths of the Mediterranean exhibit average compound-day WBTs exceeding $25\,^\circ$C, a critical threshold for human health^56,57^, with some coastal regions surpassing $26\,^\circ$C. The pattern of extreme WBT closely follows the pattern of topography in the region, with larger changes at lower elevations and smaller changes at higher elevations (Fig. 6d). Even after GHG-induced warming, high elevation areas are still too cool to cause heat stress. This expansion in spatial coverage of high WBT values in 2023 suggests a compounding effect of marine and terrestrial extremes that elevate human exposure risk across densely populated Mediterranean areas.

Together, these results highlight the amplifying effect of marine heatwaves on the persistence of coastal terrestrial heat extremes through enhanced moisture and heat coupling at the land–sea interface.

## Conclusions

Exposure to extreme heat events both on land (terrestrial heatwaves) and ocean (marine heatwaves) pose a significant threat in coastal areas in Europe, where increasing human populations and vulnerable natural ecosystems could face significant risks from these compound occurrences. Using satellite-based sea surface temperature (OISST) and station-based temperature observations (E-OBS), we show a non-linear and accelerating increase in exposure time to compound marine–terrestrial heatwave days across European coastlines over the past two decades. In 2023 the Andalusian coast (Spain), the Cantabrian Coast, Western Black Sea Coast, Western Greece and Ionian coast, and the Turkish Mediterranean coast experienced over 50 days of compound coastal marine–terrestrial heatwave. A spatial comparison between stand-alone terrestrial heatwave days and compound heatwave days reveals a growing predominance of compound events in shaping extreme heat exposure along coastal regions.

Our analysis show that the Mediterranean – a known climate change hotspot^58–62^ – exhibits the steepest increase in coastal compound marine–terrestrial heatwave days, reaching nearly 78 days by 2022, more than doubling the values observed in the early 21st century. Attribution analysis reveals that greenhouse gas (GHG) forcing accounts for approximately 95% (5–95% CI: 93–100%) of the risk associated with the 2022 event, with the CESM1-LE model estimating its probability of occurrence under a no-GHG-forcing scenario as less than 5% (5–95% CI: 7–0%). The fraction of attributable risk value for the 2023 event, with 72 days of exposure, is 0.78 (CI: 0.78–0.88), indicating that GHG forcing accounts for 78% of the risk of occurrence. The fraction of attributable risk values near 1 (100%) imply that GHG forcing is a necessary cause of such an event, signifying the strong potential to reduce their occurrence through targeted GHG mitigation.

To understand the role of GHG forcing in driving the observed nonlinear acceleration of compound coastal heat extremes, we assess how GHG emissions reshape the return periods of compound heatwaves. The response is particularly pronounced, with GHG forcing reducing return periods by up to 92 years – corresponding to a substantial increase in occurrence probability. At the 100-year FixGHG threshold, the same compound event recurs every 8 years under ALL forcing. Notably, the exposure time increases nonlinearly under GHG forcing: the rarer the event in a no-GHG scenario, the more pronounced its frequency amplification under anthropogenic forcing. This reflects a disproportionate rise in the likelihood of the most extreme compound events, emphasizing the growing risk under continued GHG emissions. This finding is consistent with theoretical and modeling studies showing that rare extremes respond disproportionately to global warming^63,64^.

Our results show that coastal marine heatwaves can amplify terrestrial heatwave exposure by up to 3.5 times, transforming short-lived terrestrial heatwaves into prolonged episodes of extreme heat and humidity. Ratios exceeding 3 are found along the western Mediterranean-including southern Spain, southern France, and western Italy-indicating that coastal heatwave exposure in these regions lasts more than three times longer when accompanied by marine heatwaves in the adjacent sea than when occurring independently. Compound heatwave events frequently push land-based wet-bulb temperature ($\ge$ $25.5^\circ$C) and near-surface specific humidity ($\ge$ 19 g/kg) beyond critical thresholds along Mediterranean coasts, indicating a clear shift toward humid heatwave regimes in coastal regions. Our findings reveals that marine heatwaves play a critical role in amplifying coastal heat extremes by strengthening moisture and heat coupling at the land–sea interface, underscoring the urgency of targeted adaptation and resilience strategies.

While this study emphasizes the influence of ocean conditions on coastal heat extremes, recent research indicates that land-derived processes can also modulate marine thermal environments. Estuarine freshwater discharge, river inflow, and catchment-driven stratification changes have been shown to modify coastal temperature dynamics and even affect the onset and duration of marine heatwaves in semi-enclosed and river-dominated systems^65–67^. These emerging land-to-sea feedbacks demonstrate that coastal thermal extremes are shaped by bidirectional interactions, rather than a one-way influence from ocean to land. Integrating both pathways is therefore essential for improving prediction, impact assessment, and adaptation planning in vulnerable coastal zones.

## Methods

### Defining compound marine–terrestrial heatwaves

Extreme heat days, over both land (daily near-surface temperature, $T_{\text {2m}}$) and ocean (sea surface temperature, SST), are defined as days when temperature exceeds a seasonally varying threshold, taken as the 90th percentile of the 1983–2012 climatological distribution. Heatwave events are identified when such exceedances persist for at least five consecutive days, allowing interruptions of fewer than two days^6,68–71^. Both terrestrial and marine heatwaves are defined using a five-day duration threshold to ensure temporal consistency when detecting compound events. Using a common 5-day criterion therefore captures sustained atmospheric–ocean interactions relevant for compound coastal events and avoids combining events with mismatched temporal scales.

Compound marine–terrestrial heatwaves are defined as periods during which both $T_{\text {2m}}$ and SST simultaneously exceed their respective thresholds in adjacent coastal grid cells along the European coastline. We focus on exposure to compound marine–terrestrial heatwaves, measured as the annual total number of compound heatwave days. Co-occurring heatwave days are defined as those on which a marine heatwave fully encompasses a terrestrial heatwave, based on paired coastal land and adjacent ocean grid cells. Results are limited to areas where E-OBS data coverage is available for terrestrial temperatures.

### Identification of coastal marine–terrestrial grid-cell pairs

To identify coastal locations where marine and terrestrial heatwaves can co-occur, we construct a land–sea adjacency mask based on the ocean (SST) and atmospheric (T2m) grids. Coastal atmospheric grid cells are identified using a 4-connected neighborhood approach. Specifically, the atmospheric mask are shifted one grid cell north, south, east, and west, and grid cells that are classified as land in the original mask but adjacent to ocean in at least one shifted mask are retained as coastal points. For each identified coastal atmospheric cell, the corresponding latitude and longitude coordinates are extracted, and the nearest marine grid cell from the SST field are paired with it using a nearest-neighbor selection (method =“nearest”). Each coastal land grid cell are therefore matched to a single unique ocean grid cell, ensuring a one-to-one pairing and avoiding any duplication. In some cases, depending on coastline geometry, multiple land cells could share the same adjacent ocean cell. This procedure produced a set of land–sea grid-cell pairs representing the coastal interface. These coastal pairs are subsequently used to compute marine heatwave days, terrestrial heatwave days, and their temporal overlap, enabling the calculation of compound coastal marine–terrestrial heatwave days.

### Compound heatwave ratio

To quantify the relationship between compound marine–terrestrial heatwaves and stand-alone terrestrial heatwaves, we calculate Compound Heatwave Ratio (CHR; Eq. 1), which measures how often compound marine–terrestrial heatwaves occur relative to terrestrial heatwaves that happen alone (without a concurrent marine heatwave).

Compound Heatwave Ratio (CHR):

1 $$\begin{aligned} {\textbf {CHR}} = \frac{\textit{Compound Marine--Terrestrial Heatwaves}}{\textit{Stand-alone Terrestrial Heatwaves}} \end{aligned}$$

### Extreme event attribution

We apply an extreme event attribution approach^43,72^ to evaluate the influence of greenhouse gas (GHG) forcing on the exposure time of compound coastal marine–terrestrial heatwave days. For this purpose, we use single-forcing experiments from the the Community Earth System Model Large Ensemble (CESM1-LE)^35^. This includes a 20-member ensemble with ALL external forcings (representing the actual world, including evolving GHG concentrations and all other natural and anthropogenic forcings) and a separate 20-member ensemble where the time evolution of GHG forcing is constant (FixGHG; a counterfactual world in which GHG concentrations are held constant). The differences between the two 20-member ensembles stem from internal variability, and each of the 20 simulations can be viewed as a plausible representation of how the real world might evolve^73^.

We quantify the co-occurrence rates of terrestrial and marine heatwaves along the European coastline as simulated in CESM1-LE by analyzing paired coastal land daily near-surface temperature ($T_{\text {2m}}$) and adjacent ocean daily sea surface temperature (SST) data. The same compound heatwave detection approach is applied consistently to both observational datasets and climate model simulation to ensure methodological comparability. A compound heatwave day is defined as a day when a marine heatwave fully encompasses a terrestrial heatwave in the corresponding paired grid cells.

We focus on the period 2000–2021, pooling the exposure time of detected compound heatwaves from each year across 20 ensemble members of the LE-CESM1 model. This provides 440 model years (22 years $\times$ 20 members) for each simulation group - 440 years for the ALL forcing simulations and another 440 years for the ALL-but-GHG (LE-FixGHG) simulations. The probability of the threshold (exposure time of compound days) being exceeded in a “counterfactual world” without GHG forcing is denoted as $P^{days}_{fixGHG}$, and the probability of exceeding the threshold (exposure time of compound days) in an “actual world” with ALL forcing is denoted as $P^{days}_{ALL}$. To quantify the uncertainty in estimated exceedance probabilities we applied a non-parametric bootstrap resampling approach with 1000 iterations. The estimated probabilities serve as the basis for computing attribution metrics; the Fraction of Attributable Risk (FAR, Eq. 2) and Probability Ratio (PR, Eq. 3) as follows:

2 $$\begin{aligned} FAR&= 1 - \frac{P^{\text {days}}_{\text {fixGHG}}}{P^{\text {days}}_{\text {ALL}}} \end{aligned}$$

3 $$\begin{aligned} PR&= \frac{P^{\text {days}}_{\text {ALL}}}{P^{\text {days}}_{\text {fixGHG}}} \end{aligned}$$

- **Fraction of Attributable Risk (FAR):** The FAR metric (Eq. 2) expresses the probability that the event would not have happened without GHG forcing. In essence, FAR quantifies the degree to which GHG forcing can be considered a necessary driver of the extreme events^74,75^.
- **Probability Ratio (PR):** The PR (Eq. 3) metric indicates how many times more likely an event is to occur under ALL forcing compared to the ALL-but-GHG (LE-FixGHG) scenario.

#### Uncertainty estimation

Sampling uncertainty was quantified using a 1000-member non-parametric bootstrap. For both the factual (ALL-forcing) and counterfactual (FixGHG) ensembles, we generated 1000 bootstrapped datasets by sampling with replacement from the original data. For each bootstrap replicate, the probability of exceeding each threshold was recalculated for both climates, yielding 1000 bootstrap estimates of the event probability under factual and counterfactual conditions, and thus 1000 corresponding FAR and PR values. The 5–95% confidence intervals were derived from the 0.05 and 0.95 quantiles of these bootstrap distributions.

At very high thresholds, the FAR values saturate at 1 because FAR cannot exceed its upper bound by definition. Consequently, when the estimated FAR approaches 1 at thresholds above $\sim$80 days (Fig. 3c), the uncertainty interval collapses to a point and appears artificially narrow. This narrowing does not reflect reduced uncertainty, but rather the constraint imposed by the bounded FAR metric. A similar behavior occurs for the PR values (Fig. 3d): once the event becomes extremely rare or entirely absent in the counterfactual climate, the PR diverges toward infinity, and the upper bound of its confidence interval cannot be meaningfully represented.

### Return level and period estimation using GEV

To estimate the return levels of compound heatwave days, we employed a non-parametric bootstrapping approach in combination with the Generalized Extreme Value (GEV) distribution, which is commonly used in climate extreme value analysis. First, annual values of the number of compound heatwave days were extracted for two scenarios: one with fixed GHG forcing (FixGHG) and one with all historical forcings (ALL). After removing missing values, we performed 1000 bootstrap resamplings of the FixGHG data. For each resampled dataset, we fitted a GEV distribution using maximum likelihood estimation. Return levels corresponding to 5-, 10-, 20-, 50-, and 100-year return periods were calculated using the inverse cumulative distribution function. This produced a bootstrap distribution of return levels for each return period. We reported the median return level and the 95% confidence intervals (2.5th and 97.5th percentiles) based on the bootstrap samples. Next, we estimated the equivalent return periods in the ALL forcing scenario for the return levels previously calculated in FixGHG. Bootstrapping was repeated on the ALL dataset to estimate the uncertainty in these return periods.

### Observation

To identify marine heatwaves (MHWs), we utilize high-resolution (0.25°or $\sim$ 28 km) daily gridded sea surface temperature (SST) data from the NOAA OISSTV2.0 dataset. This data, derived from the Advanced Very High-Resolution Radiometer (AVHRR) satellite, covers the period from 1982 to 2023^32^. To detect terrestrial heatwaves, we use E-OBS^33^ daily gridded land-only observational dataset over Europe derived from in-situ observations with 0.25°or $\sim$ 28 km resolution. Daily minimum/maximum temperature ($T_{\text {min}}$/$T_{\text {max}}$), surface specific humidity (q), and daily maximum wetbulb temperature estimated from $T_{\text {max}}$, dew point temperature (Tdew) and surface pressure, are derived from ERA5^52^ reanalysis over Europe with 0.25°resolution. The ocean evaporation is from the Objectively Analyzed Air-Sea Fluxes (OAFlux) dataset with a spatial resolution of $1^\circ$ from 1979 to 2021^51^.

## Model data

We utilize daily sea surface temperature (SST) data, and daily 2m temperature from the Community Earth System Model Large Ensemble (CESM1-LE)^35^. This includes a 20-member ensemble with all external forcings (ALL) and a separate 20-member ensemble where the time evolution of greenhouse gas (GHG) forcing is held constant (FixGHG). The differences between the two 20-member ensembles stem from internal variability, and each of the 20 simulations can be viewed as a plausible representation of how the real world might evolve^73^. The CESM1-LE simulations span the period from 1920 to 2100, using the Representative Concentration Pathway 8.5 (RCP8.5^76^) from 2006 onward. To our knowledge, CESM1-LE is the only comprehensive model currently available that provides large ensemble simulations with daily SST output for both historical and single-forcing scenarios (ALL-but-GHG, ALL-but-AER simulations). Large sample sizes are needed for event attribution in order to obtain reliable estimates of the probabilities of rare events.

## Supplementary Information

Supplementary Information.

## Data Availability

The NOAA OISST dataset is available at http://www.esrl.noaa.gov/psd/data/gridded/data.noaa.oisst.v2.html. The EOBS station-based data is available at https://surfobs.climate.copernicus.eu/dataaccess. The ERA5 data sets are available at https://www.ecmwf.int/en/forecasts/datasets/reanalysis-datasets/era5. The OAFlux data are available at https://climatedataguide.ucar.edu/climate-data/oaflux-objectively-analyzed-air-sea-fluxes-global-oceans. The CESM1 Large Ensemble (LE) data is available at https://www.earthsystemgrid.org/dataset.

## Code availability

Our heatwave analysis was conducted using the R package heatwaveR (v0.4.6;^77^) and the Python toolbox marineHeatWaves (v0.15.0;^71^), available at https://github.com/ecjoliver/marineHeatWaves.
