# Supplementary material for: Compound coastal marine–terrestrial heatwaves associated with humid-heat stress in Europe
Source: Sci Rep. 2025 Dec 12;15:43810. doi: 10.1038/s41598-025-32049-z (PMC12706064; doi:10.1038/s41598-025-32049-z)
Supplement: Supplementary file 1 — Supplementary Information. [file 41598_2025_32049_MOESM1_ESM.pdf]

Supplementary material
Compound coastal marine–terrestrial heatwaves associated with humid-heat stress in Europe

Armineh Barkhordarian[1,*], **Eline Brunet**[1,2], **and Johanna Baehr**[1]

[1]Institute of Oceanography, Universität Hamburg, Hamburg, Germany
[2]Institut Universitaire Européen de la Mer (IUEM), Université de Bretagne Occidentale (UBO), France
[*]armineh.barkhordarian@uni-hamburg.de

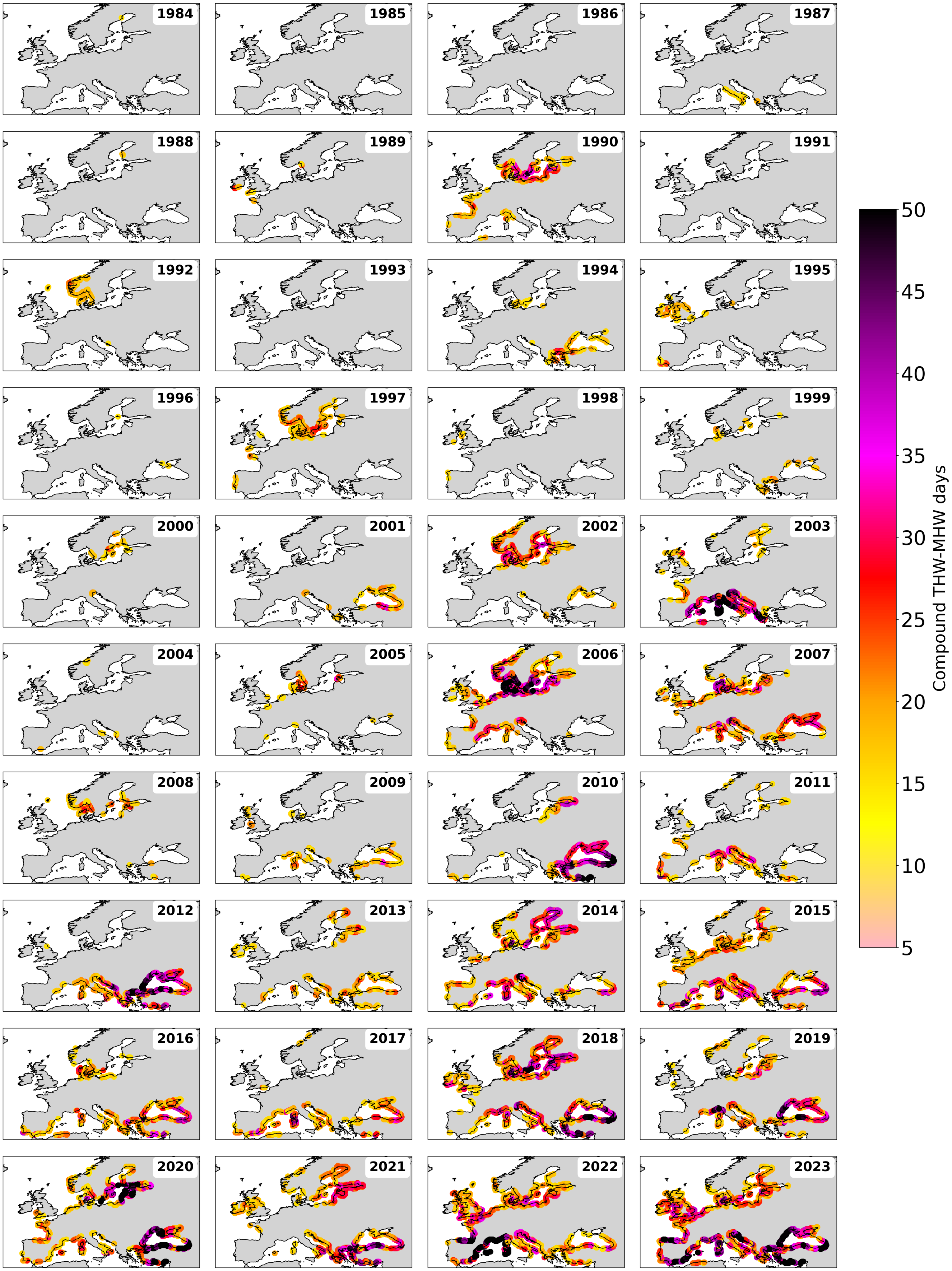

Figure 1. Observed annual spatial distribution of compound coastal marine–terrestrial heatwave (MHW–THW) days from 1984 to 2023, based on OISST and E-OBS observational records.
